# Antibacterial potency and fluoride release of a glass ionomer restorative material containing different concentrations of natural and chemical products: An *in-vitro* comparative study

**DOI:** 10.4317/jced.54606

**Published:** 2018-04-01

**Authors:** Hanaa Elgamily, Omaima Ghallab, Hoda El-Sayed, Maha Nasr

**Affiliations:** 1 Restorative and Dental Materials Department, Oral and Dental Division, National Research Centre, Giza, Egypt; 2 Operative Dentistry Department, Faculty of Dentistry, Ain-Shams University, Cairo, Egypt; 3 Dairy Science Department, Food Industries and Nutrition Division, National Research Centre, Giza, Egypt; 4 Pharmaceutics and Industrial Pharmacy Department, Faculty of Pharmacy, Ain –Shams University, Cairo, Egypt; 5 Pharmaceutics and Pharmaceutical Technology Department, College of Pharmaceutical Sciences, Mutah University, Jordan

## Abstract

**Background:**

This study investigated the antibacterial efficacy against Streptococcus mutans and fluoride release of a conventional glass ionomer (GI) contained natural and chemical agents.

**Material and Methods:**

Two hundred and ten GI specimens were divided into ten groups (n=21) according to the concentrations of the additives as; Propolis extract containing GI (Groups 1, 2, 3) with concentrations of 0.25%, 0.75% and 1.25% respectively, Miswak extract containing GI (Groups 4, 5, 6) and Chlorhexidine containing GI (Groups 7, 8, 9) with the same concentrations. The prepared specimens were subjected to antimicrobial activity by well diffusion, bacterial adherence, and fluoride release (from 2 to 72 hours) assessments.

**Results:**

A higher statistically significant antibacterial activity was found in (Groups 2, 3) compared to (Groups 8, 9), while (Groups 1, 4, 5, 6, 7, 10) no antibacterial efficacy was reported. For (Groups 2, 3) had a higher statistically significant anti-adherence effect compared to the other tested groups. Enhanced ascending increase in fluoride release was observed for (Groups 3, 4) compared to (GI).

**Conclusions:**

The increased concentration of propolis extract had a synergistic effect on the antimicrobial activity of the tested GI. Additive concentrations of 0.25% Miswak and 1.25% propolis could enhance the fluoride-releasing ability of the tested GI.

** Key words:**Propolis, miswak, chlorhexidine, glass ionomer, fluoride.

## Introduction

Despite the wide distribution of fluoride application especially in developed countries, dental caries remains one of the chronic diseases prevalent in humans worldwide (1-3). In developing countries due to the lack of facilities for dental treatment such as water and electricity for using dental handpieces and rotary burs, a manual instrumentation and atraumatic restorative treatment (ART), was elaborated (4). Lately, glass ionomer cements (GI) with enhanced physical strength were developed for ART but the ineffectiveness in complete removal of carious infected dentin, recurrent caries under GI restorations could produce (5-7). In addition, the previously dental literature showed a weak influence of GI releasing fluoride either on the profile growth of bacteria or the bacterial destruction (8). The possibility of microleakage and the limitations of GI’s physical properties, lead to increase the risk for recurrent caries (7). Integrating antibacterial agents into glass ionomer materials might consequently, be acquired a therapeutic advantage. Lastly, researchers tried to modulate GI filling materials by adding chlorhexidine (CHX) and quaternary ammonium compounds (9). Some of dental literatures proved the increased antibacterial effect of CHX containing GI materials (10,11). However, an optimal concentration of CHX which was not clearly identified as reported in many literatures and recommended to avoid any compromise of the mechanical and physical properties of the set cement (11-13). Anywise, use of natural agents against selected oral pathogens has been examined. Propolis, that’s a natural product extensively used in the traditional medication from a long time ago, has proven its effectiveness against several types of bacteria and there are numerous products containing propolis on the world market inclusive of toothpaste and mouth rinses. Little investigations on the antibacterial efficacy of propolis using different concentrations were assessed against oral bacteria (14,15). Furthermore, the use of plant extracts is one of the antibacterial approaches was investigated to improve caries control (16). In the Middle East, the most common traditional mean for oral hygiene is a chewing stick that is called miswak. Several studies reported the antibacterial activity of Miswak and its extracts against cariogenic bacteria (17,18). Therefore, it is interesting to incorporate different concentrations of CHX, Ethanolic Propolis Extract and Aqueous Miswak Extract separately into GI to investigate; (1) their inhibitory activity and anti-adherence influence on *Streptococcus mutans* (ATCC 25175) and (2) their effect on the fluoride release comparing to GIC per se.

## Material and Methods

1. Preparation of plant extract

- Ethanolic Extract of Propolis (EEP)

Propolis powder was obtained from a honey bee Egyptian supplier (Emtenan health shop, Cairo, Egypt). Thirty grams of propolis powder previously cooled (at 4°C in a dark container) were mixed with 300ml of 5% ethanol (1:10 w/v) and magnetically stirred for one hour, then stored for 48 hours at 4°C. The storage mixture was centrifuged at 2000 rpm for 15 minutes, and then filtered with Whatman No. 4 (pore size, 0.45 μm). Finally, the previous filtered liquid was kept in freezer at -20°C for 24hours then lyophilized in a lyophilizer under 5mm Hg pressure at -50°C (19,20).

- Aqueous extract of Miswak

Salvadora persica plant specimens (Miswak sticks) from the Arak tree roots (Tybah Sewak, Madinah, KSA) were utilized. The collected plant sticks were left to dry at room temperature for 10 days, then they were cut into small pieces and powdered using a commercially available food blender (21). Three hundred ml of sterile distilled water was added to 30 grams of the previously prepared Miswak powder, then the mixture was magnetically stirred, centrifuged, filtered and lyophilized as previously mentioned in Ethanolic extract of Propolis.

2. Incorporation of ethanolic Propolis extract, aqueous Miswak extract, and chlorhexidine (CHX) separately into conventional glass ionomer (GIC) restorative material 

Glass Ionomer Ionofil Plus (IP) (VOCO, GmbH, Cuxhaven, Germany) was selected for this research and provided in the form of separate powder and liquid bottles.

- Preparation of Plant extract -containing Glass Ionomer restorative material

Propolis and miswak aliquots in Glass Ionomer (IP) powder, used in three different concentrations 0.25%, 0.75% and 1.25% w/w were prepared by trituration. Propolis and miswak in the form of powder were accurately weighed on a sensitive digital balance (Sartorius, Germany), followed by addition of equivalent weight of Glass Ionomer powder in a porcelain mortar. The powder was homogeneously mixed, followed by portion-wise addition of the whole amount of Glass Ionomer powder.

- Preparation of CHX-containing Glass Ionomer restorative material

Similarly, aliquots of Chlorehexidine Digluconate liquid (CHX) (Sigma Aldrich, Steinheim, Germany) was added to the polyalkenoic acid liquid component of Glass Ionomer (IP) in the form of three concentrations; 0.25%, 0.75% and 1.25% (v/v). Accurate volumes of (CHX) were taken by a micropipette (Gilson, UK) in a glass bottle, followed by the addition of polyalkenoic acid to create the aforementioned concentrations.

The incorporation of the plant extracts and (CHX) separately into Glass Ionomer Ionofil Plus restorative material was performed at the Department of Pharmaceutics and Industrial Pharmacy, Faculty of Pharmacy, Ain-Shams University, Cairo, Egypt.

3. Preparation of all groups of Conventional Glass Ionomer restorative material specimens

For preparing Glass Ionomer (IP) (control) specimens, one scoop of powder to one drop of liquid was mixed on a glass plate according to manufacturer’s recommendation (mixing ratio: 4.7-5.6 g of powder: 1 g of liquid). While for preparing Glass Ionomer material containing plant extract either EEP or Miswak specimens: one scoop of previously prepared Glass Ionomer (IP) powders containing one of the plant extracts with each selected concentration, was mixed to one drop of Glass Ionomer (IP) liquid. For preparing CHX-containing Glass Ionomer material specimens: one scoop of Glass Ionomer (IP) powder was mixed to one drop of previously prepared Glass Ionomer (IP) liquids containing CHX with the same three concentrations.

4. Study design 

A total of two hundred and ten specimens of experimentally prepared Glass Ionomer restorative material were divided according to the type and concentrations of the additives that were incorporated into the Glass Ionomer (IP) as the following; Propolis extract containing Glass Ionomer (IP) (n=63) (groups: G1:GI+Propolis(0.25%), G2:GI+Propolis(0.75%), G3:GI+Propolis(1.25%)) (n=21 for each group); Miswak extract containing Glass Ionomer (IP) (n=63) (groups:G4:GI+Miswak(0.25%), G5:GI+Miswak(0.75%), G6:GI+Miswak(1.25%)) (n=21 for each group); and CHX containing Glass Ionomer (IP) (n=63) (groups:G7:GI+CHX(0.25%), G8:GI+CHX(0.75%), G9:GI+CHX(1.25%) (n=21 for each group) and a group of Glass Ionomer (IP) without additive as a control (n=21).

5. Testing procedures

- Antimicrobial test

*Streptococcus mutans* ATCC 25175 type strain (16S rRNA gene, Serotype c. carious dentin) was obtained from (Microbiological Resources Centre, MIRCEN, Cairo, Egypt) and used throughout the study. Bacteria were cultured overnight at 37°C in the Brain Heart Infusion Broth (BHI, Merck KGaA 64271 Darmstadt, Germany) and used as inoculums. The turbidity of the suspension was adjusted to the McFarland 0.5 turbidity standard (Densimat, BioMerieux, France). At this absorbance, the concentration of bacteria is standardized to about 1x CFU/ml and used as a working microbial solution (22).

Bacterial Inhibition Test

The antimicrobial activity of the three different concentrations (0.25%, 0.75% and 1.25%) of each plant extract and CHX containing Glass Ionomer (IP) restorative material was assessed using well diffusion method. Twenty µl of the previously prepared working microbial solutions was spread evenly over a trypticase soy agar plate (TSA, Difco, USA). The experimental Glass-Ionomer (IP) restorative material was mixed directly inside the wells with the powder/liquid ratio as described above, the mixing time was 30-40 seconds, the working time was 2.5-3.5 minutes and the setting time (at ambient temperature) was 5-6 minutes. Five wells measuring 4 mm in diameter were made in each plate, three of these wells were filled with one of the selected concentration of each testing additives and the fourth well was filled with a Glass Ionomer (IP) without additive (control) (n=7 plates per group). While the fifth one that was filled with 50 μl of 0.2% chlorhexidine digluconate as a positive control against* S. mutans* (23). After incubation of the plates at 37 ᵒC for 24 hours, the zones of bacterial growth inhibition around the wells were measured in mm unit.

Adherent/planktonic bacterial count test

A total of sixty-three specimens representing all groups (G1-G9) (n=7 per group) were performed by using cylindrical molds (10 mm in diameter and 2 mm in thickness). Powder/Liquid ratio was mixed in accordance to the manufacturer instructions as previously mentioned for each group. After setting, each specimen was dipped separately into the test tube containing Tripticase Soy Broth media (TSB; Merck KGaA 64271 Darmstadt, Germany) supplemented with 5% (w/v) sucrose. Twenty µl of previously prepared suspension containing *S. mutans* ATCC 25175 (1x 10 -6 CFU/ml) was inoculated in each previous test tube and one test tube was left as a control without specimen. All testing tubes were incubated at 37 o C temperature for 24hours, while positioned at a 300 angle from the horizontal to increase the surface area for bacterial adherence. At the end of the incubation period, the TSB media, which contained non-adherent bacteria, was decanted, and the tubes were gently washed with 0.5 ml of saline. The decanted broth and the washes were pooled, centrifuged, and suspended in saline. The adhering bacteria to the glass were removed by 0.5 M NaOH, centrifuged, and suspended in saline (24,25). Each suspension containing non-adherent bacteria and adherent bacteria of each group separately was vortexed for two minutes and sonicated for one minute.

Then, dilutions of 1x 10 -2 and 1x 10 -3 of saline containing previously vortexed and sonicated suspension were performed (22). Thirty µl of each dilution was spread on the surface of selective media Mitis Salivarius Bacitracin (MSB; BD Difco, France) plate. After incubation period of 24- 48 hours, the suspension containing either adherent or non-adherent (planktonic) bacteria were counted to determine the number of colony forming unit per ml (CFU/ml).

- Measurements of fluoride ions release

Seven specimens for each group were prepared using a split Teflon mold with a diameter of 5 mm and a thickness of 1mm, with powder/liquid ratio as described by the manufacturer. After setting, each specimen was placed in 10 ml deionized water (pH ~ 7) and stored at 37°C. The specimens were transferred to new vials with renewed deionized water for 2, 6, 12, 24, and 72 hours measuring times. Accompanying the transition to new vials, the old deionized water was stored at -20°C in order to perform all fluoride concentration analyses in one session. The specimens were filtered on Merman filter paper then measured on the Ion Chromatography ICs 5000+ SP (Thermo scientific, USA) which consisting of a P680 pump, an automated sample injector. Data collection and processing were performed with a personal computer equipped with Dionex Chromeleon software. The measurement unit is mg/l or ppm. The specimens were analyzed at (Water Pollution Research Department, Environmental Research Department, National Research Centre, Egypt).

6. Statistical analysis

Data were explored for normality using Kolmogorov-Smirnovand Shapiro-Wilk tests. For parametric data; One-way ANOVA followed by Tukey post-hoc test was used to compare between more than two groups in non-related samples. For non-parametric data; Kruskal Wallis test was used to compare between more than two groups in non-related samples. Mann Whitney was used to compare between two groups in non-related samples. The mean and standard deviation values were calculated for each group in each test. The significance level was set at *P* ≤ 0.05. Statistical analysis was performed with IBM® SPSS® Statistics Version 20 for Windows.

## Results

1. Inhibition zones results

**Figure 1:**
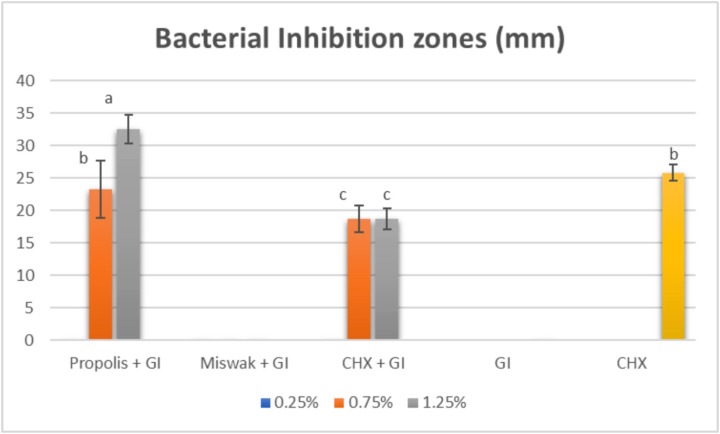
Bar chart representing the means of inhibition zones (mm) for different concentrations in all tested groups.

According to well diffusion test results, the means of the inhibition zones diameter (mm) for the studied groups against *S. mutans* were displayed in (Fig. 1).

The size of inhibition zones of GI containing propolis were obviously dependent on the three different concentrations (0.25%, 0.75%, and 1.25%) of Propolis where (*P*≤0.001).The highest statistically significant mean value was found in (G3 1.25%) (32.60 ± 2.22) compared to (G2 0.75%) (23.30 ± 4.45) and CHX per se (control group) (25.80±1.23), while in (G1 0.25%), it did not exhibit any antibacterial efficacy against *S. mutans* as well as the three concentrations of GI containing Miswak in (G4 0.25%), (G5 0.75%) and (G6 1.25%). For GI containing CHX, the concentration 0.75% (G8) and 1.25% (G9) showed an inferior statistically significant inhibitory activity against *S. mutans* compared to (G2), (G3) where (*P*≤0.001). The control group Glass ionomer (GI) did not show any antibacterial efficacy against *S. mutans*.

2. Bacterial count results

- The Planktonic Bacterial count

**Figure 2:**
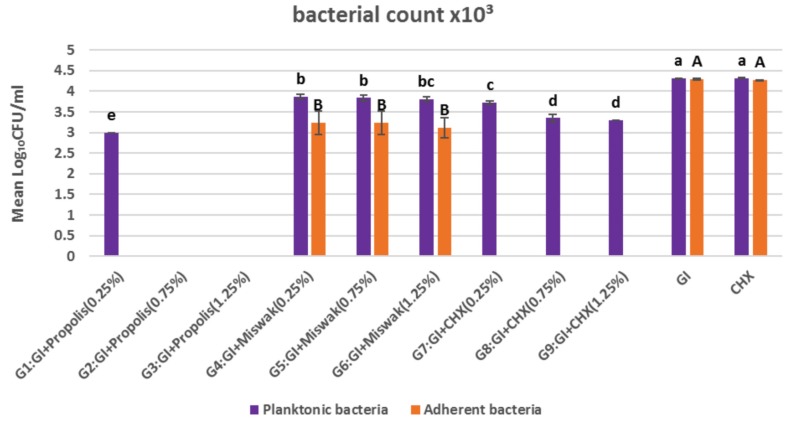
Bar chart representing the means of planktonic and adherent bacterial count (CFU/ml) for all tested groups.

The effect of different additive’s concentrations in each group regarding the planktonic bacterial count of *S. mutans* was represented in (Fig. 2). For GI containing propolis groups, a lower statistically significant mean CFU/ml value of planktonic bacterial count reported for (G1) compared to the other additives groups. While in (G2), (G3), lack of CFU/ml of planktonic bacteria was noticed.

For (G4), (G5) and (G6) of GI containing Miswak, there was no statistically significant difference between the mean values of CFU/ml of planketonic bacteria (3.87±0.06, 3.84±0.07, 3.81±0.06) respectively, where *P* value=0.4.

For the Groups of CHX containing GI, (G7) showed a higher statistically significant mean value of CFU/ml of planketonic bacteria (3.72±0.04) compared to (G8) and (G9) (3.35±0.09, 3.30±0.00) where *P* value= 0.008. There was no statistical significant difference between (G8) and (G9).

Moreover, (GI) and CHX control groups showed higher statistically significant mean values of CFU/ml of planktonic bacterial count compared to the other groups.

- Adherent Bacterial Count:

The Propolis containing GICs groups (G1, G2, G3) recorded lack of CFU/ml of adherent bacterial count. While Miswak containing GI groups (G4, G5, G6) exhibited no statistically significant difference regarding the mean values of adherent bacterial count (3.24±0.028, 3.24±0.28, 3.12±0.24) respectively where *P* value =0.4. CHX containing GI groups (G7, G8, G9) showed absence of adherent bacterial count. GI and CHX control groups reported higher statistically significant mean values of adherent bacterial count compared to the other groups (4.30±0.02, 4.27±0.01) respectively and there was no statistical significant difference between them.

3. Fluoride release results

**Figure 3:**
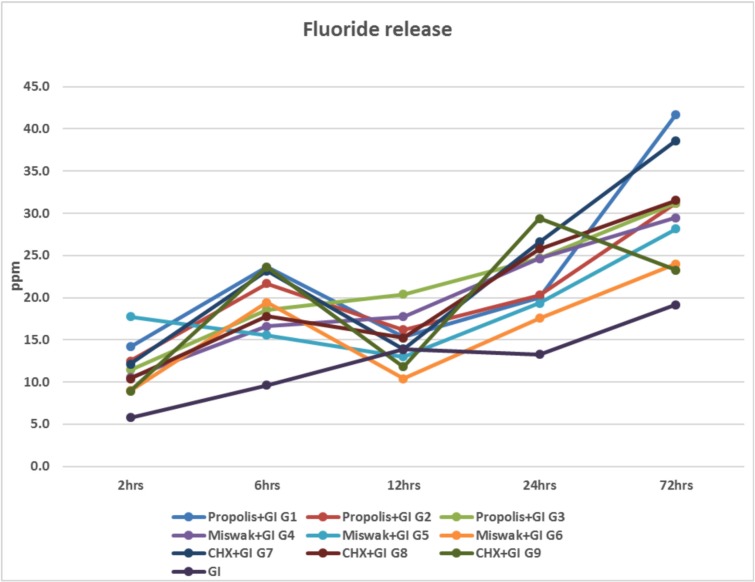
Line chart representing the mean values of fluoride release (ppm) from 2 hours till 72 hours measuring times for all the tested groups.

In (Fig. 3), the mean values of fluoride release (ppm) for (G1), (G2), (G5), (G6), (G7), (G8) and (G9) showed an increase from 2 hours measuring time till 12 hours, then it decreased after reaching 12 hours. Finally, the mean values of fluoride release elevated again till reaching 72 hours. While for (G3), (G4) and conventional glass ionomer control group (GI), the mean values of fluoride release increased from 2 hours measuring time till reaching 72 hours without decreasing at any time of measurements.

## Discussion

Caries ailment still remains a noteworthy general medical issue regardless of the boundless utilization of fluoride and the decrease in caries commonness saw in the larger part of exceedingly industrialized countries (3). The utilization of Glass Ionomer cements (GI) as an antibacterial remedial material for the treatment of caries is dicey on account of the possible microleakage and impediments related with their physical properties (7). However, annual scientific research reported that the main cause for GI failure is still the recurrent caries (26-28). Therefore, combining bactericidal agent with Glass Ionomer materials would provide an alternative approach. Overtures, numerous trials for the enhancement of the antibacterial characteristic of GICs have been documented by means of the addition of antibacterial solutions like chlorhexidine (CHX) (29,30). Notwithstanding, an ideal convergence of CHX was prescribed to keep away from any trade off of the mechanical and physical properties of the set cement (12,13). The use of plant extracts is one of the many approaches investigated for caries control (16). Miswak extract is a medicinal plant that reported to have anti-plaque and many other pharmacological properties (31). Also, it is cheap and its taste is agreeable and not unpleasant. As well, Propolis extract which is a natural non-toxic substance produced by the honeybee appears a modern choice as it is inexpensive and available without difficulty and also, possessed many biological therapeutic activities (32). With regard to previously mentioned knowledge, three remarkable antimicrobial agents; chlorhexidine digluconate (CHX), ethanolic extract of Propolis (EEP), and aqueous Miswak extract were selected for this in-vitro study in the form of three different concentrations and incorporated into conventional GI restorative material.

*S. mutans* is a solitary bacterial biofilm and has been utilized as a part of a few past investigations of antibacterial dental materials as a result of its involvement with dental caries (33-36). Therefore, *S. mutans* was chosen for the present study to screen the antibacterial activity of each natural antimicrobial agents incorporating into GI at three different concentrations (0.25%, 0.75%, 1.25%) using agar plate diffusion method.

Agar plate diffusion was a preferable method in the current research due to its facilities to investigate the unset materials with a massive range of specimens and also, it is less expensive. Unfortunately, the inhibition zone observed through that test does not represent any data about the viability of the examined microorganisms as it could not be able to differentiate between the bacteriostatic and the bactericidal effects (12). Moreover, the in-vitro check does not confer with the clinical reality wherein various species of bacteria may be developing in complex biofilms.

As it was reported in the literature that, the materials had appreciably more antibacterial effect for the unset state than the completely set one (38). This could be attributed to the initial bactericidal efficacy of the most dental materials while setting and their low pH throughout this time might too have an impact. Therefore, this current study used the unset conventional GI material to be examined with the well diffusion method based on the previous findings (12).

According to well diffusion test results (Fig. 1), the tested groups of GIC containing EEP (G1, G2, G3) showed that, the inhibition zones diameters that were created against *S. mutans* growth have been virtually based upon the concentration of the EEP introduced into the GI. This previous observation validated that the addition of EEP to Glass ionomer Ionofil Plus (IP) led to a restorative material that had improved antibacterial properties over the traditional glass ionomer per se concerning *S. mutans* growth. However, the findings of this study were in contrast to Nursen Topcuoglu *et al.* (22); who reported that diameters of inhibition zones which were determined against *S. mutans* were not based upon the concentration of EEP. The difference could be attributed to the significant variations between the laboratories; the determination of inhibition zones values was also linked to the inherent virulence of bacteria and their susceptibility (39).

While there were no inhibition zones exhibited by the tested groups of GI containing Miswak and revealed no statistically significant difference between them; (G4 0.25%), (G5 0.75%) and (G6 1.25%). The latter result could be due to incorporation of three different low concentrations into the GI which were ineffective. This was in agreement with a previous study conducted by Kabil *et al.* (40); who demonstrated that the antimicrobial effect increased significantly by adding higher concentrations of Miswak into GI.

Preceding researches the usage of traditional GI tested conflicting outcomes about the antibacterial effect determined through the addition of CHX; some pronounced that antimicrobial activity was based upon the concentration of the disinfectant delivered to GI (10,4,30), whilst others indicated no dose-response impact (1,42). The results in the current study revealed a higher statistically significant difference between the concentrations of 0.75% (G8) and 1.25% (G9) compared to the concentration of 0.25% (G7) as in (Fig. 1), and this would correlate the elution rate of the antibacterial agent from the GI with a specific concentration, where synergism has been appeared to happen between the metal particles and the cationic CHX antibacterial agent (30).

Regarding to the adherent bacterial count assay, this present study described the development of an assay based on the ability of each plant extract and CHX containing GI to inhibit adherence of *Streptococcus mutans* (25175) to glass surfaces. Using a smooth glass surface in this study to symbolize the hard surface of the tooth and prove to be equally fine as adherence model compared to hydroxyapatite or a tooth surface (43). Adhesion of cariogenic streptococci to the smooth surfaces of teeth and restoration surfaces is a very essential level within the pathogenesis of dental caries. This adherence can mimic in-vitro and needs sucrose within the growth media, which acts as the principle direction that helps bacterial adherence to surfaces. *S. mutans* have the ability to convert the sucrose into glucans which reinforce the adhesion and consequently gives a share in dental biofilm formation. As documented that, the adherent bacteria are greater resistant than their planktonic forms in biofilms, in-vitro assay could simulate the clinical testing of anti-plaque property of different restorative materials (22).

The results in (Fig. 2) showed that EEP containing GI had a statistically significant anti-adherence effect on S. mutans compared to the other tested groups. *S. mutans* serotype c is especially hydrophobic, and that hydrophobic bonding regarded to be a crucial element of their adherence activities. It is consequently advised that anti-adherence activities of EEP containing GI as shown in the results could have been altered this natural hydrophobic bond which in the midst of the bacteria and the smooth glass surfaces (44). While the lower anti-adherence activities of CHX containing GIC than EEP containing is probably a result of the material wastage via elution. Another explanation, as has been advised with the aid of Ribeiro and Ericson (10); the lower in CHX is associated with the formation of insoluble salts with the GI. The groups of Miswak containing GI as shown in (Fig. 2), no statistically significant difference in CFU/ml of adherent bacteria was reported between (G4), (G5) and (G6) as well as for planktonic bacteria, while a statistically significant difference was found between each of them and all other groups. This previous finding could be due to the active volatile components of Miswak extract in GI might be lost during its preparation (45). Therefore, the adherence inhibition assay was shown to be effective and reproducible in quantitating the ability of each plant extract and CHX incorporating into GI to prevent the adherence of *S. mutans* (25175 serotype c) to the smooth glass surfaces.

The mean values of fluoride release results in (ppm) unit of Glass Ionomer specimens after the addition of different concentrations of plant extract (EEP, Miswak) and CHX individually is presented in (Fig. 3). The results reported that, the incorporation of 0.25% (G1) and 0.75% (G2) into GI elevated the release of fluoride at 6 hours measuring time. After GI specimens setting, it decreased after reaching 12 hours, finally fluoride elevated in release again till reaching 72 hours. While the concentration of 1.25% of EEP in GI (G3) increased in release from two hours measuring time till reaching 72 hours without decreasing at any time of measurements. This latter finding could be due to the physical presence of EEP in the matrix of GIC that might help in creating a route for the emission of fluoride. It also could be explained by the increased solubility of GIC by adding EEP, which might give rise to the fusillade release of fluoride in the early 12 hours after specimens setting (8). Therefore, the experimental GI with EEP had improved the antimicrobial properties of GI with conservation the retentive characteristics of fluorine ion release when compared to conventional GICs per se.

Regarding the results of Miswak containing GI (G4, G5, G6), the selected concentrations exhibited increase of fluoride release at the different times of measurement except for (G5, G6), which showed a decrease in fluoride release at 12 hours measuring time followed by increase until reaching 72 hours. It was obviously noticed that addition of Miswak with different concentrations had increased the fluoride release compared to the conventional GI per se. This might be due to the ability of Miswak to release fluoride. It was in contrast to several studies, where they reported that Miswak extract could release fluoride but it was considered unlikely, as it is soluble and fluoride total content in the Miswak, especially that released when soaked in water, is negligible (< 0.07 μg/ml) (46-48). At 12 hours measuring time, fluoride release was decreased, this might be due to the chemical reaction between fluoride and any of the organic or inorganic substances present in Miswak extract that protected or inhibited the release of fluoride as suggested by Morch and Bjorvatn (49).

It was observed that, the fluoride release for (G7), (G8) and (G9) of CHX containing GI was lower at 12 hours measuring time, then it increased till reaching 72 hours. This might be explained Hoszek and Ericson (13); who found that the association amongst fluoride and the cationic CHX, bringing about the precipitation of salts with bring down dissolvability, leaving fluoride less accessible during the setting of the conventional GI.

In conclusion, within the limitations of this study and the persistent changes of the oral environment, the following conclusions could be drawn: The increased concentration of propolis extract had a synergistic effect on anti-microbial activity of the tested conventional GI. As well, the additive concentrations of 0.25% Miswak and 1.25% propolis could enhance the fluoride releasing ability of the tested conventional GI.
